# Identification of important extracellular vesicle RNA molecules related to sperm motility and prostate cancer

**DOI:** 10.20517/evcna.2021.02

**Published:** 2021-04-08

**Authors:** Yu Zhang, Ning Ding, Shenmin Xie, Yaqun Ding, Mengna Huang, Xiangdong Ding, Li Jiang

**Affiliations:** National Engineering Laboratory for Animal Breeding, Key Laboratory of Animal Genetics, Breeding & Reproduction, Ministry of Agriculture, College of Animal Science & Technology, China Agricultural University, Beijing 100193, China.

**Keywords:** Seminal plasma extracellular vesicles, transcriptome, sperm motility, prostate cancer, immune cells

## Abstract

**Aim:**

Many male diseases are associated with sperm quality, such as prostate cancer (PCa), oligospermia, and asthenospermia. Seminal plasma extracellular vesicles (SPEVs) play important roles in sperm function. In this study, we explored the specific RNA molecules in SPEVs that play an important role in sperm motility and found promising biomarkers of PCa in SPEVs.

**Methods:**

Pigs have become an ideal model for human biomedical research. In this study, the whole transcriptome profiles of SPEVs of boars with high or low sperm motility were studied for the first time. Important long non-coding RNAs, microRNAs, and genes were identified through differentially expressed analysis and weighted correlation network analysis (WGCNA). In addition, we established a diagnosis model of PCa by differentially expressed miRNAs homologous with human.

**Results:**

In total, 27 differentially expressed miRNAs, 106 differentially expressed lncRNAs, and 503 differentially expressed genes were detected between the groups. The results of WGCNA show one module was significantly associated with sperm motility (r = 0.98, *FDR *= 2 × 10^-6^). The value of highly homologous miRNAs for the diagnosis of PCa was assessed and the combination of hsa-miR-27a-3p, hsa-miR-27b-3p, hsa-miR-155-5p, and hsa-miR-378a-3p exhibited the highest sensitivity (AUC = 0.914). Interestingly, mRNA expression of SPEVs was mainly enriched in resting memory CD4 T cells and monocytes, and 33 cell marker genes of monocytes overlapped with the differentially expressed genes.

**Conclusion:**

These data demonstrate that SPEVs of individuals with high and low sperm motility exhibit distinct transcriptional profiles, which provide valuable information for further research on diagnosis and molecular mechanism of diseases.

## INTRODUCTION

In humans, some male diseases, such as prostate cancer (PCa), oligospermia, and asthenospermia, are associated with poor sperm motility and function^[1,2]^. Sperm motility is one of the important indicators of sperm quality. Due to similarities in the metabolic characteristics, cardiovascular system, and proportional size of organs between pigs and humans, particularly the high similarity between the pig and human genome sequences, pigs have become an ideal model for human biomedical research^[3,4]^. In addition, the pig model provides the advantages of low genetic variation and alleviation of human-specific confounding factors (such as smoking and drinking)^[5]^. Therefore, studying complex human diseases using pig models is a good strategy.

Extracellular vesicles (EVs), including exosomes (with an average size of ~100 nm), are double-layered phospholipid membrane vesicles that are released by most cells^[6]^. Many studies have shown that EVs contain DNA, RNA, metabolites, lipids, and proteins^[7]^. As carriers of bioactive molecules and important intercellular signaling molecules, EVs can execute many biological functions and are considered to be of great significance^[8]^. Several studies have been devoted to the identification of effective clinical biomarkers in EVs, particularly microRNAs, lncRNAs, and proteins^[9-11]^.

Semen is composed of sperm and seminal plasma, which contains a large number of EVs. Seminal plasma EVs (SPEVs) are derived from a mixture of fluids secreted by the organs of the testis, epididymis, and accessory glands^[12]^. A previous study showed that seminal plasma plays an important role in the morphological changes and maturation of sperm^[13]^ and participates in the metabolism, survival, and transportation of sperm in the female reproductive tract^[14]^. In recent years, some studies have demonstrated that SPEVs play an important role in sperm function, such as sperm motility^[15]^, sperm capacitation, and acrosome reaction^[16]^. It has been reported that the noncoding RNAs contained in SPEVs are involved in the protection of sperm from the female immune response triggered by contact with sperm in the female reproductive tract^[17,18]^. Moreover, SPEVs can combine with sperm *in vitro*, promote sperm movement, prolong the effective survival time of sperm, improve the integrity of the sperm plasma membrane, and increase the antioxidant capacity^[19]^. However, the specific RNA molecules in SPEVs that play an important role in sperm motility remain unclear.

miRNAs are endogenous single-stranded 18-25 nt small noncoding RNAs capable of regulating gene expression at the posttranscriptional level^[20]^. It has been reported that miRNAs participate in the regulation of many genes involved in reproductive biology, such as germ cell development, maturation, and fertilization^[21,22]^. miRNAs in human SPEVs have recently been studied by RNA sequencing and microarray technology^[23]^. For instance, Abu* et al*.^[24] ^(2016) suggested that 7 and 29 miRNAs are expressed at high and low levels, respectively, in seminal exosomes of oligoasthenospermia patients compared with those of normal individuals. In addition, Barcelo* et al*.^[25] ^(2018) compared the miRNA expression profiles of seminal exosomes from patients with different pathological types of azoospermia (obstructive and secretory azoospermia) and identified five miRNAs (miR-182-3p, miR-205-5p, miR-31-5p, miR-539-5p, and miR-941) as potential biomarkers of patients with secretory azoospermia.

The potential application of miRNAs in EV-based liquid biopsy has attracted extensive attention. Some important miRNAs in EVs that are related to sperm quality and PCa have been identified by comparing patients with healthy controls^[26,27]^. However, most of these EVs originate from human urine^[10,28,29]^ and blood plasma^[30]^, and only a few studies have found several specific miRNA markers in human semen exosomes^[31]^. EVs from seminal plasma can better reflect the condition of sperm and prostate tissue. Therefore, highly homologous miRNAs identified in pig SPEVs that are related to sperm quality can provide valuable information for the early diagnosis of patients with PCa.

This study constituted the first study of the whole transcriptome of SPEVs of Yorkshire boars with different sperm motility levels. Differential miRNA, gene, and lncRNA expression profiles were identified between the different groups, and the differentially expressed lncRNA (DEL)-differentially expressed miRNA (DEmi)-differentially expressed gene (DEG) regulatory network was constructed. Several important miRNAs, lncRNAs, and genes affecting sperm motility were identified. In addition, the value of highly homologous miRNAs for the diagnosis of PCa was assessed, and an ROC analysis showed that hsa-miR-24-3p, hsa-miR-27a-3p, hsa-miR-27b-3p, and hsa-miR-23b-3p could be used as promising biomarkers for PCa diagnosis.

## METHODS

### Ethics statements

All protocols for the collection of semen samples of all animals were approved by the Institutional Animal Care and Use Committee at China Agricultural University (Permit number: DK996). The experiments in this study were conducted according to regulations and guidelines established by this committee.

### Animals and samples

The sperm motilities of 230 large white boars were measured using a computer-assisted sperm analysis (CASA) system (IVOS Ⅱ, France) from a national boar station. The sperm motility performance of three consecutive semen samples from each boar was evaluated. Eleven boars with extremely high or low sperm motility were selected for SPEV extraction. All individuals were sexually mature, with an age between 14 and 36 months. Detailed information of the 11 boars is shown in Supplementary Table 1.

### Analysis of sperm motility

One ejaculate from each boar was collected using the gloved-hand method. Specialized professionals obtained the sperm-rich fractions of ejaculates from each individual, and the sperm motilities of these samples were assessed using the CASA system (IVOS Ⅱ, France). Then, 950 µL of preheated diluent were added to 50 µL fresh semen and mixed gently. After 5 min of incubation at 37 °C, 7 µL of sperm suspension were placed on a prewarmed glass slide and covered with a glass coverslip. The glass slides were examined with a bright field under an optical microscope at a total magnification of 200×. The percentage of motile sperm was estimated in five different microscopic fields for each sample using the CASA system.

### Experimental design

According to the sperm motilities of boars, the eleven individuals were divided into two groups: the H group and the L group. The boars in the H group had a higher total sperm motility (> 0.97), whereas the boars in the L group had a lower total sperm motility (< 0.73). Detailed information is shown in Supplementary Table 1.

### Isolation of SPEVs 

Each semen sample was centrifuged (800× *g*, 20 min at 17 ℃) for the separation of sperm and supernatant. The supernatant was used for the extraction of EVs. SPEVs were isolated by ultracentrifugation. as previously described^[32]^. Thirty-five milliliters of semen plasma from each sample were centrifuged at 10,000× *g* and 4 ℃ for 30 min to remove cellular debris and smaller pieces of undissolved seminal gel. The supernatant was then transferred to a clean centrifugal tube and centrifuged at 12,000× *g* and 4 °C for 1 h. The supernatant was then transferred to an ultracentrifugation tube (Beckman, USA) and centrifuged at 120,000× *g* and 4 °C for 1.5 h. The sediments were resuspended in DPBS (Gibco, USA), and the previous step was repeated. The sediments were then resuspended in 2 mL of DPBS and filtered through 0.22-µm filters (Millipore, USA).

### Transmission electron microscopy

Twenty microliters of SPEVs were placed on a formvar carbon-coated grid for 5 min at room temperature. The grids were washed three times with distilled water, stained with 1.0% uranyl formate (Electron microscope China, China) for 5 min, and dried for 2 min under incandescent light. The grids were observed and photographed under a transmission electron microscopy (HT770, Tokyo, Japan).

### Nanoparticle tracking analysis (NTA)

The concentrations of SPEVs were diluted to 1 × 10^6^-1 × 10^9^ particles/mL with PBS. A ZetaView PMX 110 (Particle Metrix, Meerbusch, Germany) equipped with a 405-nm laser was used to examine the size and quantity of the isolated particles. A 1-min video shot at a frame rate of 30 frames per second was used to analyze the motion of the particles using NTA software (ZetaView 8.02.28).

### Western blot analysis

SPEVs were cleaved with RIPA buffer (Solarbio, Beijing, China) containing 1% protease inhibitor on ice for 30 min. Twenty-five microliters of the protein samples were separated by SDS-PAGE and then transferred to PVDF membranes (Millipore, USA). The membrane was blocked with 5% (w/v) skim milk for 2 h, washed five times with 1× TBST, incubated with antibodies against CD9 (sc-13118, Santa Cruz, CA, USA), CD63 (sc-5275, Santa Cruz, CA, USA), Alix (sc-53, 540, Santa Cruz, CA, USA), Calnexin (10,427-2-AP, Promega, Madison, WI, USA), and Tsg101 (sc-13, 611, Santa Cruz, CA, USA) for 12 h at 4 ℃ and then with secondary antibodies for 2 h at 37 ℃, and detected with an ECL system.

### RNA extraction and RNA sequencing

Total RNA was isolated from SPEVs using a RNeasy Mini Kit (Qiagen, Germany) according to the instructions. The RNA quality was verified by 1% agarose gel electrophoresis, and the RNA concentration and integrity were measured using the RNA Nano 6000 Assay kit and the Agilent biological analyzer 2100 system (Agilent Technologies, CA, USA).

Whole transcriptome sequencing was performed to obtain insight into the types of RNAs, including miRNAs, mRNAs, and lncRNAs, in SPEVs. Small RNA and long RNA libraries were established. Long RNA libraries were generated using the SMARTer Stranded Total RNA-Seq Kit (Takara Bio Inc.) according to the manufacturer’s instructions, and the index code was added to the attribute sequence of each sample. Small RNA libraries were generated using the QIAseq miRNA Library Kit (Qiagen, Frederick, MD, USA) following the manufacturer’s recommendations, and the index code was added to the attribute sequence of each sample. The quality of the library was evaluated with an Agilent analyzer 2100 and by qPCR. TruSeq PE cluster kitv3 CBOT HS (Illumina, San Diego, CA, USA) was used to cluster the index coding samples on a cbot cluster generation system. The library of each sample was then sequenced with an Illumina HiSeq 2500 platform (Illumina, USA) to generate paired-end reads.

### RNA-sequencing analysis

For the identification of miRNAs, we first used cutadapt to trim adapter sequences of 3’ reads (AACTGTAGGCACCATCAAT) and kept the sequences with lengths between 18 and 32 nt after trimming^[17]^. To filter ncRNAs, such as rRNA, tRNA, scRNA, snRNA, and snoRNA, as well as repeated sequences, the clean data were aligned to the Silva^[33]^, GtRNAdb^[34]^, Rfam^[35]^, and Repbase databases^[36]^. The remaining sequences were further identified as miRNAs through miRDeep2^[37]^ based on the following steps: (1) alignment to the pig reference genome (ftp://ftp.ensembl.org/pub/release-97/fasta/sus_scrofa) with no mismatch; and (2) further alignment to known mature and precursor miRNA sequences downloaded from the miRbase database (v21) (http://www.mirbase.org/).

For the identification of lncRNAs, we first obtained the clean reads after removing the low-quality reads. The clean reads were mapped to the pig reference genome using Hisat2^[38]^. Reference genome and gene annotation files were downloaded from Ensembl (ftp://ftp.ensembl.org/pub). The mapped reads of each sample were assembled and merged into transcripts using StringTie^[39]^. All identified transcripts were guided by the gene models of GffCompare^[40]^. The novel transcripts were filtered according to the following steps: (1) Among the different class codes, only transcripts annotated by “i”, “u”, “x”, “o”, and “e” were retained^[41]^; (2) Transcripts with a single exon or a length shorter than 200 nt were removed^[42]^; (3) Transcripts with FPKM ≥ 0.1 were retained; and (4) Four software programs for coding potential analysis, namely CNCI^[43]^, CPC^[44]^, Pfam^[45]^, and CPAT^[46]^, were employed to predict the protein-coding ability, and the transcripts of the overlapping results obtained from these four software programs were considered candidate lncRNAs.

### Identification of differentially expressed miRNAs, genes and lncRNAs

DESeq2 was used to identify the DEmis, DEGs, and DELs based on unnormalized read counts^[47]^. We first filtered the miRNAs, genes, and lncRNAs with low expression levels. The DEmis, DEGs, and DELs were then detected by comparing the H group with the L group. For each comparison, miRNAs, genes, and lncRNAs that satisfied the criteria *P *< 0.05 and |Fold Change| (FC) > 1.5 were considered significantly differentially expressed.

### Validation of differentially expressed miRNAs

Ten DEmis (ssc-miR-142-3p, ssc-miR-146a-5p, ssc-miR-155-5p, ssc-miR-184, ssc-miR-223, ssc-miR-23a, ssc-miR-23b, ssc-miR-24-3p, ssc-miR-378, and ssc-miR-378b-3p) were randomly selected to validate the small RNA sequencing results using TaqMan advanced miRNA assays. Total RNA was extracted and purified from SPEVs of the H and L groups using the RNeasy Mini kit (Qiagen, Germany). Two microliters of total RNA from each sample were used for miRNA reverse transcription using an PrimeScript™ RT reagent Kit (Perfect Real Time) (Takara Biotechnology, China) according to the manufacturer’s recommended protocols. The same amount of *Caenorhabditis elegans* cel-miR-39 miRNA was added to each SPEV sample as an external calibration for RNA extraction, reverse transcription, and miRNA amplification. Real-time quantitative PCR (qPCR) was performed on an ABI7500 qPCR system (Applied Biosystems) with Premix Ex Taq™ (Probe qPCR) (Takara Biotechnology, China) according to the manufacturer’s instructions. Specific miRNA TaqMan expression probes (Life Tech, CA, USA) were used for miRNA quantification [Supplementary Table 2]. Each sample was analyzed in triplicate, and all miRNAs were standardized using cel-miR-39 miRNA.

### WGCNA of mRNAs and lncRNAs

We constructed DEGs and DELs coexpression networks using WGCNA (v1.12.0) implemented in R. The original RNA-seq count expression matrix containing DEGs and DELs was used as an input file, and the expression matrix was then normalized by the variance stabilizing transformation procedure implemented in DESeq2^[48]^. We used the “one step method” to divide the gene expression matrix into different modules based on pairwise Pearson’s correlation. In the one-step method, a softpower of 12 was selected as the threshold to identify the coexpressed DEGs and DELs modules. The hub DEGs and DELs within important modules were defined by an absolute value of membership greater than 0.7 and an absolute value of gene significance greater than 0.2^[49]^. KOBAS software (http://kobas.cbi.pku.edu.cn/) was used for an enrichment analysis of the hub DEGs in important modules^[50]^.

### Construction of DEL-DEmi-DEG networks

RNAhybrid was used to predict potential DELs related to DEmis with a predicted energy < -25^[51]^. miRanda and RNAhybrid were used for the prediction of potential DEGs that interact with DEmis^[52]^. The parameters used in the miRanda analysis were the following: single residue pair match scores (S) > 150 and Gibbs free energy during double-strand formation (△G) < -20 kcal/mol^[52]^. Based on the predicted regulatory DEL-DEmi and DEmi-DEG pairs, DEL-DEmi-DEG networks were constructed via shared DEmis. The results were visualized using Cytoscape 3.5.1 software^[53]^.

### Establishment of miRNAs for the diagnosis of PCa

We downloaded mature miRNA datasets of PCa from The Cancer Genome Atlas (TCGA) (https://portal.gdc.cancer.gov/). In total, 16 homologous DEmis between pigs and humans obtained after filtering DEmis with low expression were used to establish a miRNA diagnosis model for PCa. The efficacy of the candidate miRNAs or their combinations was analyzed by receiver operating characteristic (ROC) curves, and the area under the ROC curve (AUC) was calculated. First, 82 samples were used as the test set (52 cases *vs.* 30 controls) to analyze the diagnostic accuracy of 16 homologous DEmis between pigs and humans through a ROC analysis, and the AUC was also calculated. Homologous DEmis with a high AUC (> 0.7) was used to establish the miRNA diagnosis model of PCa based on the 132 samples validation set, which included 80 cases and 52 controls. The efficacy of the candidate miRNAs or their combinations was analyzed by ROC, and the AUC was calculated. In addition, the SPEV transcriptome data from eight Duroc boars and 11 Yorkshire boars were used to investigate the composition of SPEVs in different immune cell types using the CIBERSORTx tool (https://cibersortx.stanford.edu/runcibersortx.php), and 22 types of immune cells were evaluated in this study^[54]^. The marker genes of CD4+ T cells and monocytes were obtained from CellMaker websites (http://biocc.hrbmu.edu.cn/CellMarker/).

## RESULTS

### Characterization of SPEVs of Yorkshire boars

**Figure 1:**
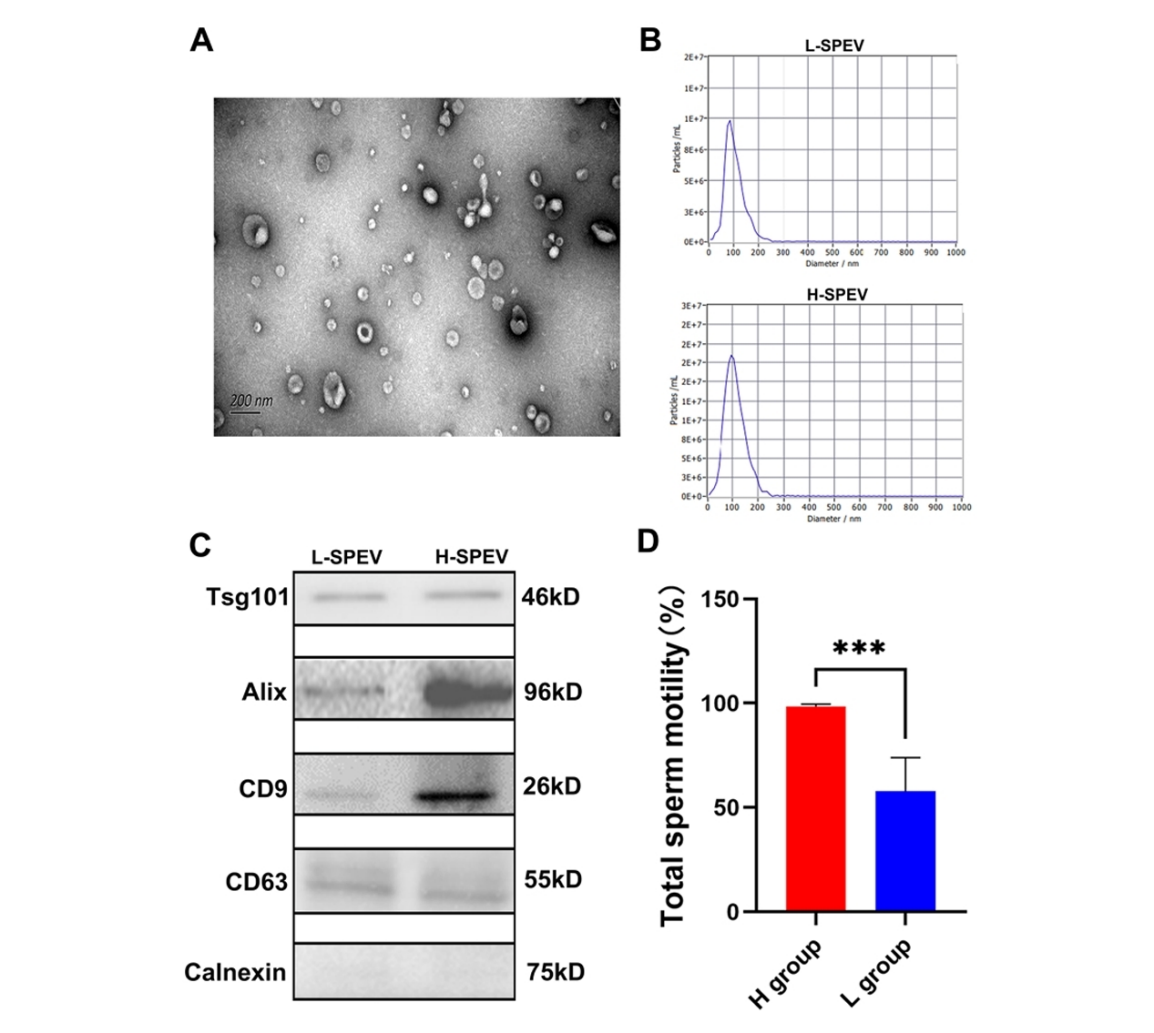
Characterization of SPEVs from Yorkshire boars. (A) TEM image of SPEVs. (B) NTA results showing that the semen-derived EVs were approximately 50-200 nm in diameter. (C) The isolated SPEVs expressed the EV markers Tsg101, Alix, CD9 and CD 63, and the negative marker Calnexin was not found in our isolated SPEV samples. D. Bar plot comparing the total sperm motility between the high-sperm-mobility (H) group and the low-sperm-mobility (L) group.

The results of transmission electron microscopy show that most SPEVs appeared intact and had the typical cup shape [Figure 1A]. The mean size of the SPEVs was 108.7 nm, and the particle size ranged from 50 to 200 nm [Figure 1B]. A Western blotting analysis detected extracellular vesicle markers (CD9, CD63, Alix, and Tsg101) in SPEVs isolated from one boar in the high-sperm-motility (H) group and one boar in the low-sperm-motility (L) group. In contrast, Calnexin, a negative marker of EVs, was absent in SPEVs from the two boars [Figure 1C]. Phenotypic analysis showed that significant differences in the total sperm motility were found between the two groups [Figure 1D].

### Summary of RNA-seq data

To obtain small RNA libraries, we acquired 333,466,614 raw data , and an average of 30,315,146 raw data was obtained from each sample. After quality control of the raw data, an average of 19,719,374 clean data was obtained from each sample, and, after the annotation of ncRNAs and repeat sequences, the average percentage of reads from each sample that were aligned to the reference genome was 77.53%. The Q30 base percentages of all samples ranged from 94.99% to 96.59%. To obtain long RNA libraries, we acquired 253.24 gigabases (Gb) of clean data, and an average of 23.02 Gb was obtained from each sample. The average Q30 base percentage of all the samples was 90.97%. Detailed information is shown in Supplementary Table 3. In addition, 203.44 Gb of clean long RNA data were obtained from our previous study of SPEVs of eight Duroc boars.

### Overall description of miRNAs of SPEVs

**Figure 2:**
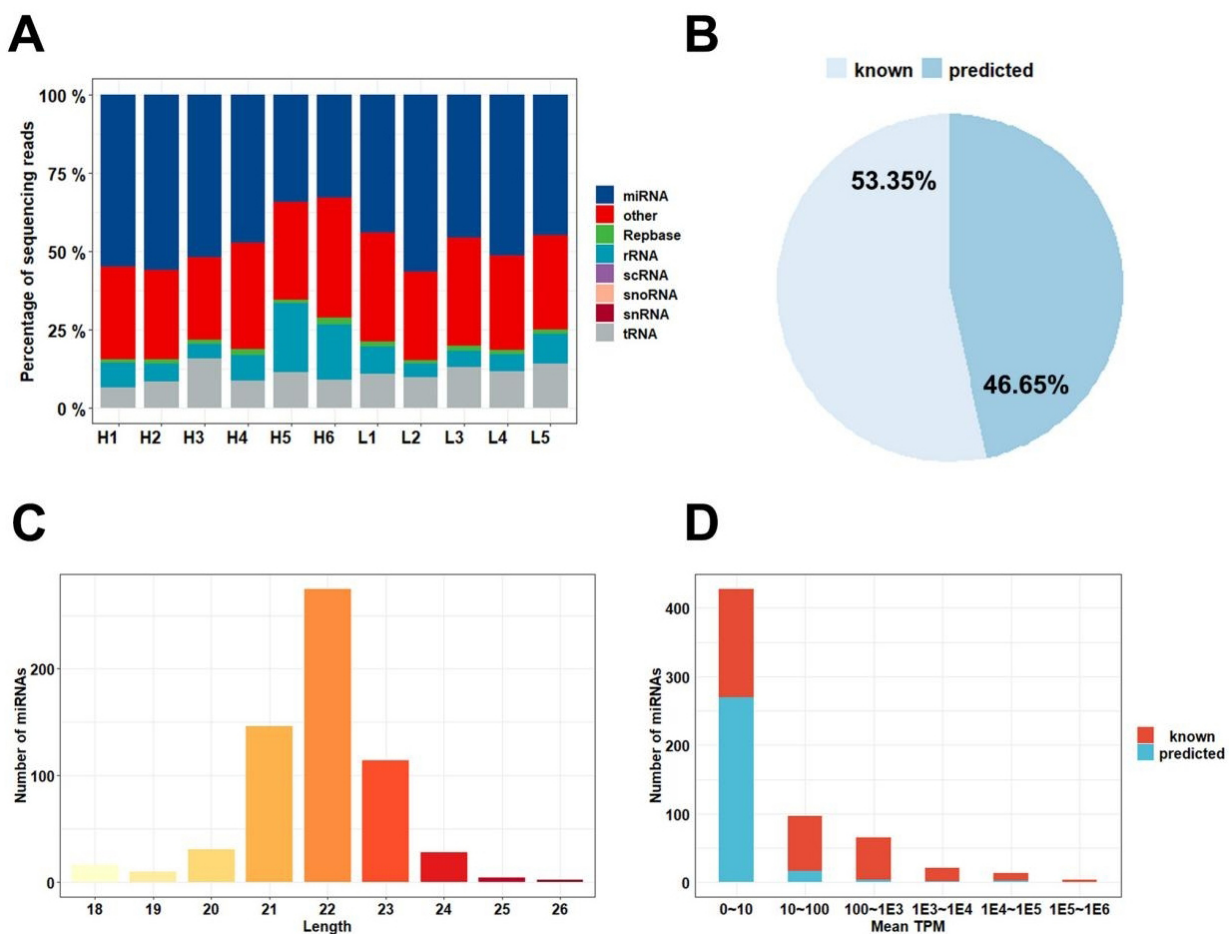
miRNA contents of SPEVs. (A) Read mapping distribution of the short noncoding RNA types in SPEVs. (B) Proportion of known and predicted miRNAs detected in pig SPEVs. (C) Distribution of the miRNA lengths. (D) Number of miRNAs with different expression levels. Most of the miRNAs were expressed at low levels, and 16 miRNAs were expressed at high levels (above 10,000 TPM).

The small RNA libraries read were mapped to the annotated miRNA database (miRBase), and many different types of small RNAs, such as miRNA, ribosomal RNA (rRNA), transporter RNA (tRNA), small nuclear RNA (snRNA), small nucleolar RNA (snoRNA), and small cytosol RNA (scRNA), were found in Yorkshire boar SPEVs [Figure 2A]. The average content of miRNAs in all the samples was 47.39%. In total, 626 mature miRNAs were detected in SPEVs, and these included 334 (53.35%) known miRNAs and 292 (46.65%) novel miRNAs [Figure 2B]. The length distribution of miRNAs in all the samples was mainly 21-23 nt [Figure 2C]. As shown in Figure 2D, the vast majority of miRNAs (83.71%) were expressed at low levels (less than 100 TPM on average), and only 16 miRNAs were highly expressed (more than 10,000 TPM on average). The 20 most highly expressed miRNAs accounted for 92.04% of all miRNA-associated expression. The top four miRNAs with the highest expression levels belonged to the let-7 family (ssc-let-7c, ssc-let-7a, ssc-let-7f-5p, and ssc-let-7e), and these accounted for 64.51% of the top 20 miRNAs [Table 1].

**Table 1:** The 20 most expressed miRNAs in SPEVs

**miRNA**	**Mean TPM**	**% of Top 20**	**% of miRNA**
ssc-let-7c	218093.9	23.69%	21.81%
ssc-let-7a	157823.7	17.15%	15.78%
ssc-let-7f-5p	119953.7	13.03%	12.00%
ssc-let-7e	97927.36	10.64%	9.79%
unconservative_5_246044	77462.69	8.42%	7.75%
ssc-miR-148a-3p	39357.21	4.28%	3.94%
ssc-miR-10a-5p	27201.52	2.96%	2.72%
ssc-miR-10b	27187.77	2.95%	2.72%
ssc-miR-125b	19533.44	2.12%	1.95%
ssc-miR-21-5p	19362.82	2.10%	1.94%
unconservative_6_269105	16654.9	1.81%	1.67%
ssc-miR-200b	15842.16	1.72%	1.58%
ssc-miR-191	14447.83	1.57%	1.44%
ssc-miR-141	13231.56	1.44%	1.32%
ssc-let-7i-5p	11030.17	1.20%	1.10%
ssc-miR-30a-5p	10835.5	1.18%	1.08%
ssc-miR-30d	9248.16	1.00%	0.92%
ssc-miR-125a	9055.415	0.98%	0.91%
ssc-miR-16	8502.95	0.92%	0.85%
ssc-miR-26b-5p	7743.076	0.84%	0.77%

### Clustered miRNAs and correlation of expression

**Figure 3:**
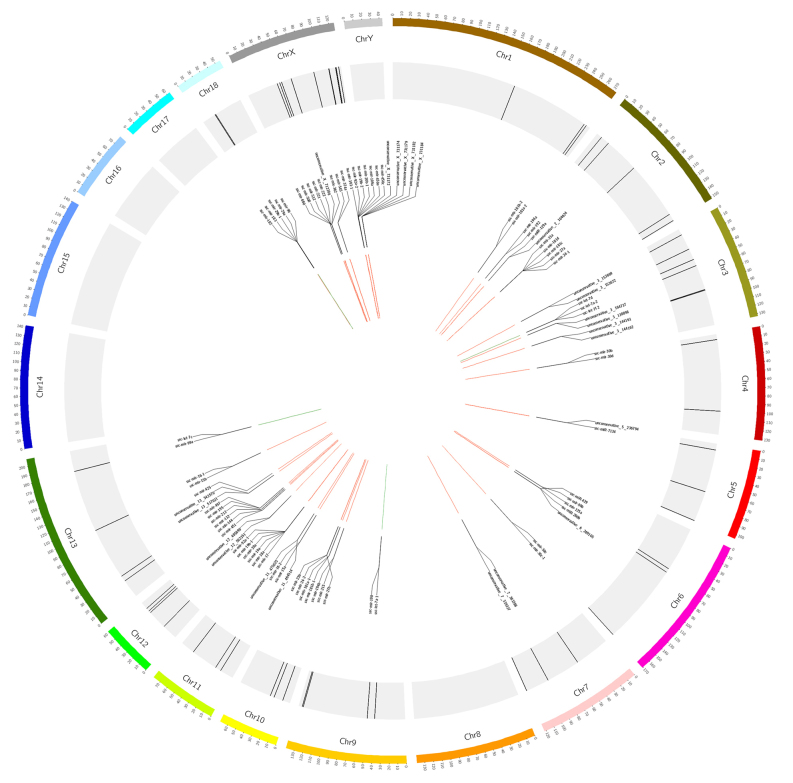
Distribution of miRNA clusters on pig chromosomes. The chromosomes are indicated on the outer circle. The genomic clusters are indicated on the middle circle by black lines. A subset of these miRNA clusters is shown on the inner circle. The correlation between miRNA pairs in the same miRNA cluster is shown in the center of the graph and the positive and negative correlations are indicated by red and green lines, respectively. All significant correlations (|r| > 0.7, *P *< 0.05) are visualized.

As recommended by miRBase, 10-kb windows were used to obtain clusters of miRNAs. All miRNAs detected in SPEVs were divided into 58 clusters [Supplementary Table 4]. In total, 163 miRNAs were included in these clusters, 42 of which were new miRNAs. To highlight genomically-clustered miRNAs that could be coexpressed, the correlations among miRNAs contained in the same cluster were calculated. Interestingly, for most clusters (40 out of 58), the miRNAs in the same cluster showed strong expression correlations (|r| > 0.7, *P* < 0.05; Supplementary Table 5). In addition, 91 miRNAs within 39 clusters showed a significantly positive correlation [Figure 3].

### Identification and validation of differentially expressed miRNAs in SPEVs

**Figure 4:**
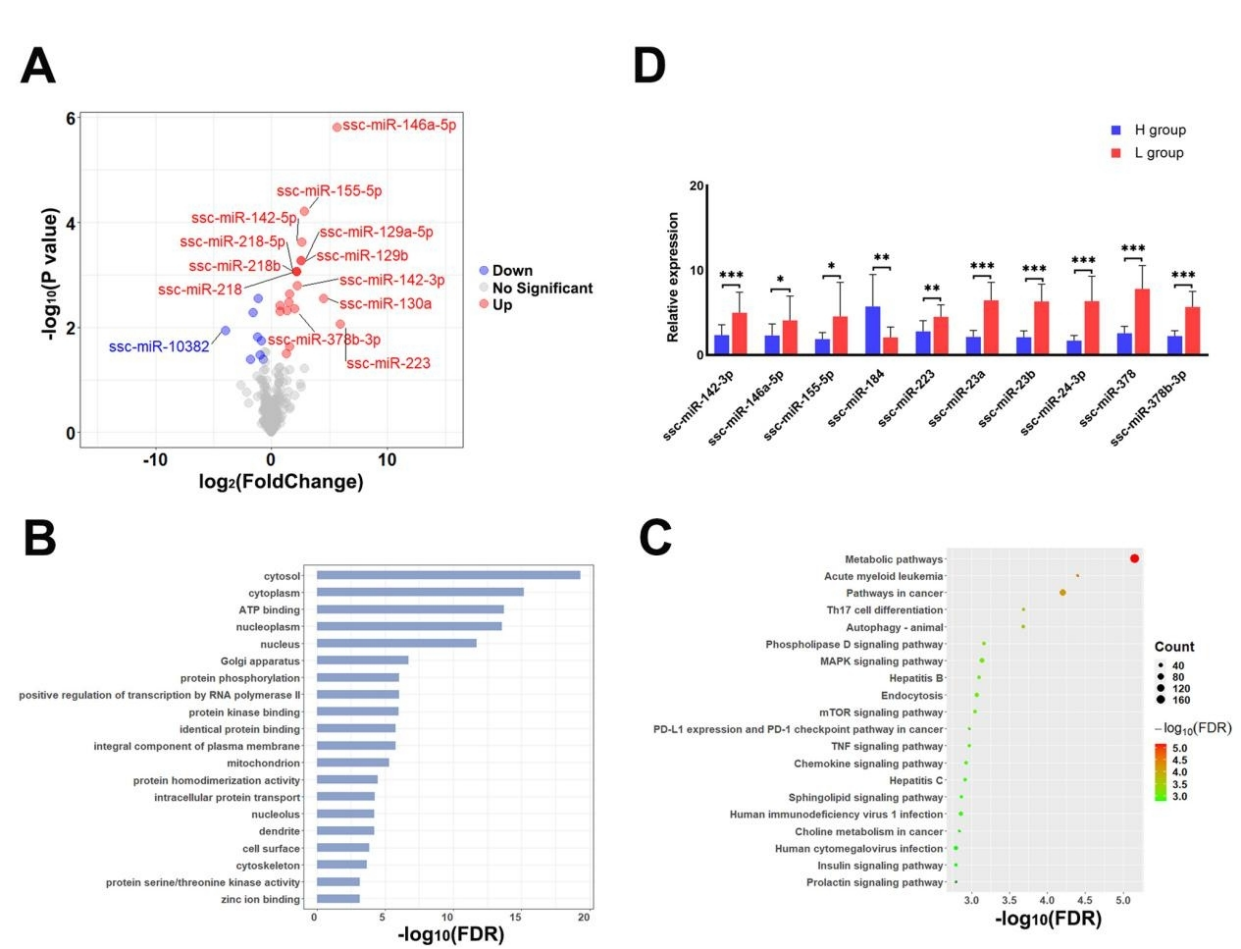
Identification, validation and functional enrichment analysis of DEmis in SPEVs. (A) Volcano plot displaying the DEmis between the SPEVs of the H and L groups. (B) Top 20 biological processes (FDR < 0.05) enriched with mRNAs targeted by DEmis. (C) The top 20 KEGG pathways (FDR < 0.05) enriched with the target genes of DEmis are shown in the bubble diagram. (D) The expression levels of ten DEmis were validated by qPCR.

In total, 27 DEmis were detected between the H and L groups [Supplementary Table 6]. Most of the DEmis (19 out of 27) showed higher expression in the boars of the L group [Figure 4A], and, among these DEmis, ssc-miR-223 exhibited the highest upregulation (log_2_FC = 5.97). To further understand the potential functions of the genes targeted by the DEmis, we performed gene ontology (GO) and KEGG pathway analyses using KOBAS. The target genes of the DEmis were predicted with miRanda and RNAhybrid, and 2579 target genes were predicted by both software programs. The enrichment analyses revealed 107 significant GO terms and 92 significant pathways related to the DEmis obtained from the comparison of the H and L groups (*FDR *< 0.05) [Supplementary Table 7]. The top 20 GO terms and pathways are shown in Figure 4B and C, respectively.

To validate the DEmis identified by small RNA sequencing, 10 DEmis were randomly selected for qPCR verification. The results show that nine miRNAs (ssc-miR-142-3p, ssc-miR-146a-5p, ssc-miR-155-5p, ssc-miR-223, ssc-miR-23a, ssc-miR-23b, ssc-miR-24-3p, ssc-miR-378, and ssc-miR-378b-3p) were significantly upregulated in the SPEVs of the L group compared with those of the H group, and one miRNA (ssc-miR-184) was significantly downregulated in the L group. These results are consistent with the small RNA-seq data [Figure 4D].

### lncRNA and mRNA expression profiles in SPEVs

**Figure 5:**
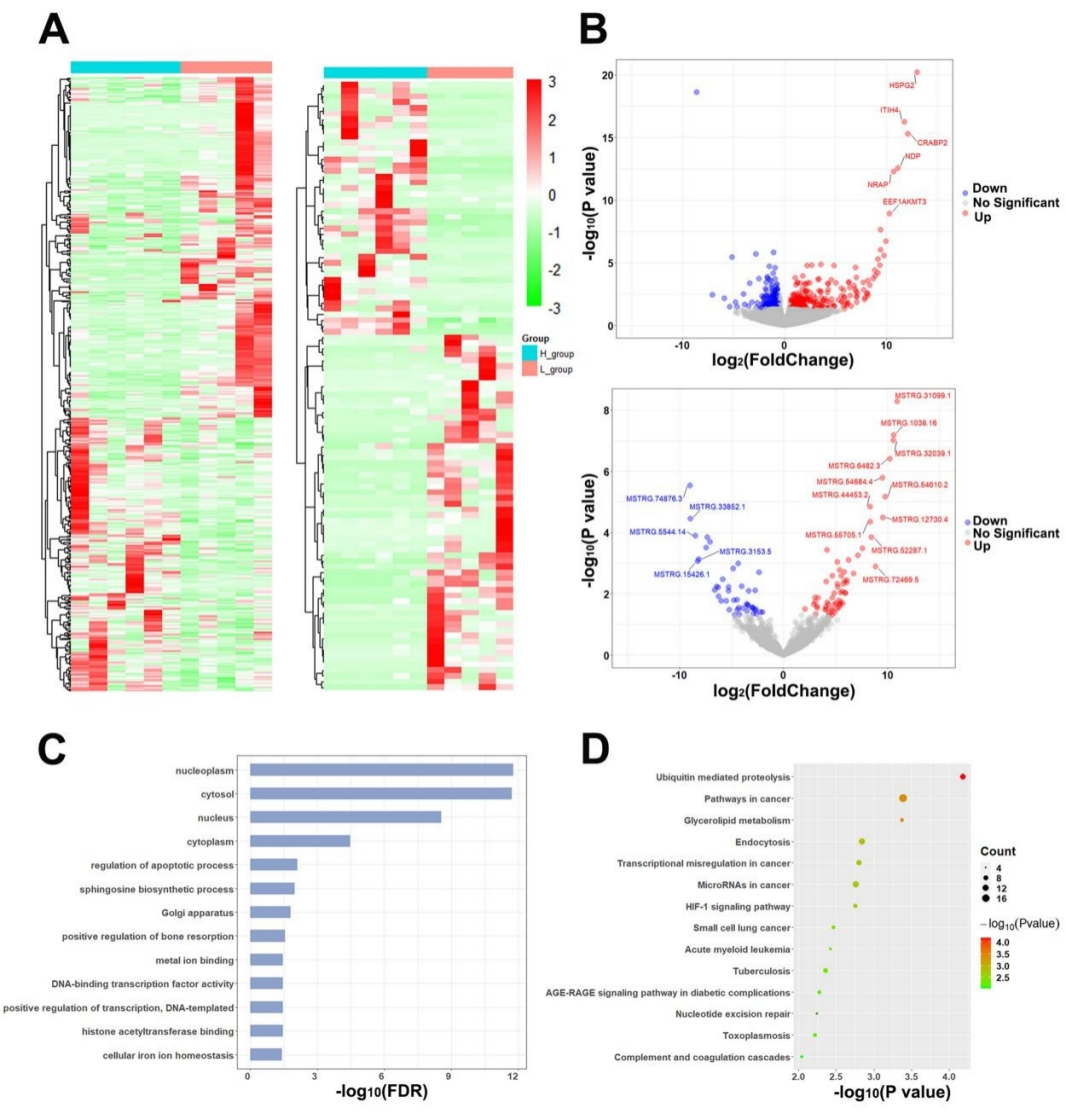
Differential expression and enrichment analysis of genes and IncRNAs in SPEVs. (A) Heatmap plots of DEGs (left) and DELs (right) across all the samples in our SPEV long mRNA dataset. (B) Volcano plot displaying the mRNAs (upper) and IncRNAs (below) showing differential expression between the SPEVs of the H group and those of the L group. (C) Bar plot showing the GO enrichment of DEGs (FDR < 0.05). (D) Bubble plot showing the KEGG enrichment of DEGs (*P *< 0.01).

In total, 503 significant DEGs [Supplementary Table 8] and 106 significant DELs were identified from the comparison of SPEVs from the H group and those from the L group [Supplementary Table 9]. The heatmap revealed differences in the expression levels of the significantly dysregulated mRNAs and lncRNAs in the samples with different sperm motility levels [Figure 5A]. Most DEGs (271 out of 503) and DELs (62 out of 106) showed higher expression in the L group than in the H group [Figure 5B], and, among these DEGs, *HSPG2* and *FEN1* exhibited the highest upregulation (log_2_FC = 13.05) and the highest downregulation (log_2_FC = -8.58), respectively. Similarly, MSTRG.31099.1 and MSTRG.74876.3 showed the highest upregulation (log_2_FC = 10.94) and the highest downregulation (log_2_FC = -9.01), respectively, among the DELs. The gene ontology analysis identified 13 significant biological process categories (*FDR *< 0.05) [Figure 5C, Supplementary Table 10]. The pathway analysis revealed that ubiquitin-mediated proteolysis, pathways in cancer, glycerolipid metabolism, endocytosis, microRNAs in cancer, and HIF-1 signaling pathway were the predominant biological processes represented [Figure 5D, Supplementary Table 10].

### Gene coexpression modules associated with sperm motility

**Figure 6:**
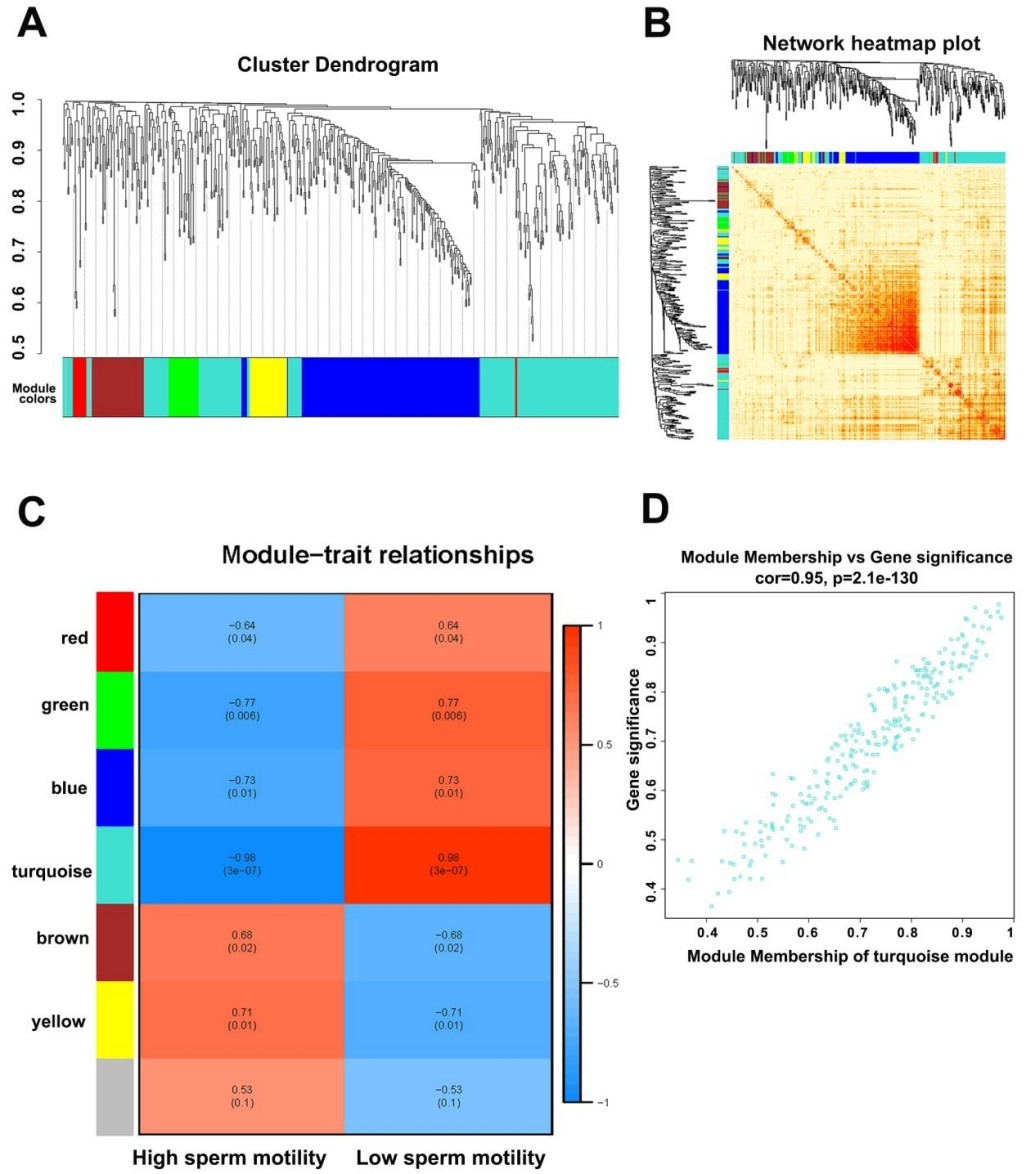
Weighted gene correlation network analysis (WGCNA) of SPEV DEGs and DELs. (A) A cluster dendrogram of the coexpression network module was produced based on the expression of DEGs and DELs. (B) A heatmap plot of DEGs and DELs in the network is shown. (C) The relationship of DEGs and DELs in various modules between the high-sperm-motility (H) and low-sperm-motility (L) group was investigated. (D) The turquoise module exhibited the highest relationship with sperm motility.

A weighted correlation network analysis (WGCNA) of all long RNA-seq data from SPEVs identified six coexpressed DEGs and DELs modules [Figure 6A], and the heatmap plot of the topological overlap matrix (TOM) is shown in Figure 6B. The DEGs and DELs in the six color modules were then continuously used to calculate their correlation with module traits. Interestingly, we found that the turquoise module, which included 202 DEGs and 54 DELs, was most significantly associated with high or low sperm motility (r = 0.98, *FDR *= 2 × 10^-6^) [Figure 6C and D]. The second most significant module was the green module (r = 0.73, *FDR *= 0.0175), which included 19 DEGs and 14 DELs [Figure 6C]. We detected hub genes in each significant module and found 374 hub DEGs and 74 hub DELs [Supplementary Table 11].

**Figure 7:**
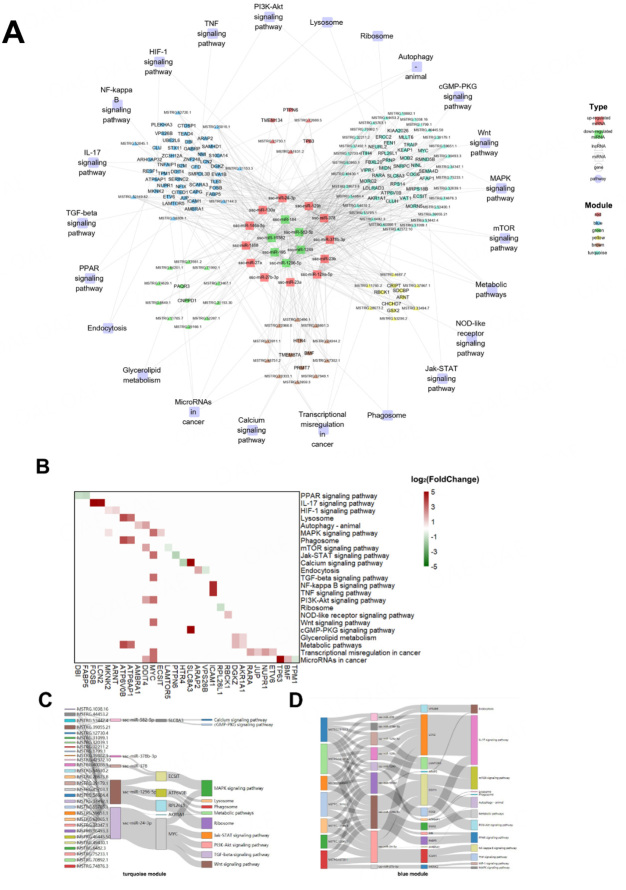
Regulatory DEL-DEmi-DEG network and relationships among DEmis, DELs and DEGs. (A) Regulatory network of DEL-DEmi-DEG pairs in pig SPEVs. The outer circle represents the important pathways related to sperm motility. The middle circle represents the six coexpression modules depicted in different colors. The inner circle displays the 12 upregulated miRNAs (red) and six down regulated miRNAs (green) in the L group. The hub DELs and DEGs are indicated by triangles and circles, respectively. The links show the DEL-DEmi regulatory relationships, DEmi-DEG regulatory relationships and DEG-pathway annotations. (B) Heatmap demonstratingthe hub DEGs annotatedin important pathways. (green: downregulated in the L group, red: upregulated in the L group). (C) DEL-DEmi-DEG interaction network of the turquoise module. (D) DEL-DEmi-DEG interaction network of the blue module.

According to the target relationship among the hub DEGs, DEmis, and DELs, the regulatory networks of SPEVs were constructed [Figure 7A]. In total, 23 pathways, such as microRNAs in cancer, glycerolipid metabolism, PPAR signaling pathway, IL-17 signaling pathway, HIF-1 signaling pathway, Jak-STAT signaling pathway, MAPK signaling pathway, mTOR signaling pathway, calcium signaling pathway, PI3K-Akt signaling pathway, and metabolic pathways, were included in the network [Figure 7A]. In total, 30 hub DEGs were annotated in these important pathways [Figure 7B]. Moreover, five DEmis (ssc-miR-582-5p, ssc-miR-378b-3p, ssc-miR-378, ssc-miR-1296-5p, and ssc-miR-24-3p) were predicted to interact with 29 hub DELs and six hub DEGs (*SLC8A3*, *ECSIT*, *ATP6B0B*, *RPL26L1*, *AKR1A1*, and *MYC*) in the turquoise module, which are involved in 11 signaling pathways [Figure 7C]. Nine DEmis, seven hub DELs, and thirteen hub DEGs in the blue module were involved in endocytosis, mTOR signaling pathway, lysosome, phagosome, metabolic pathways, NF-kappa B signaling pathway, PI3K-Akt signaling pathway, HIF-1 signaling pathway, and MAPK signaling pathway [Figure 7D]. It is worth noting that *MYC *in the turquoise module and *DDIT4 *in the blue module can be targeted by ssc-miR-24-3p, and both of these are involved in the PI3k-Akt signaling pathway [Figure 7C and D].

### SPEV-derived miRNAs as biomarkers of PCa

**Figure 8:**
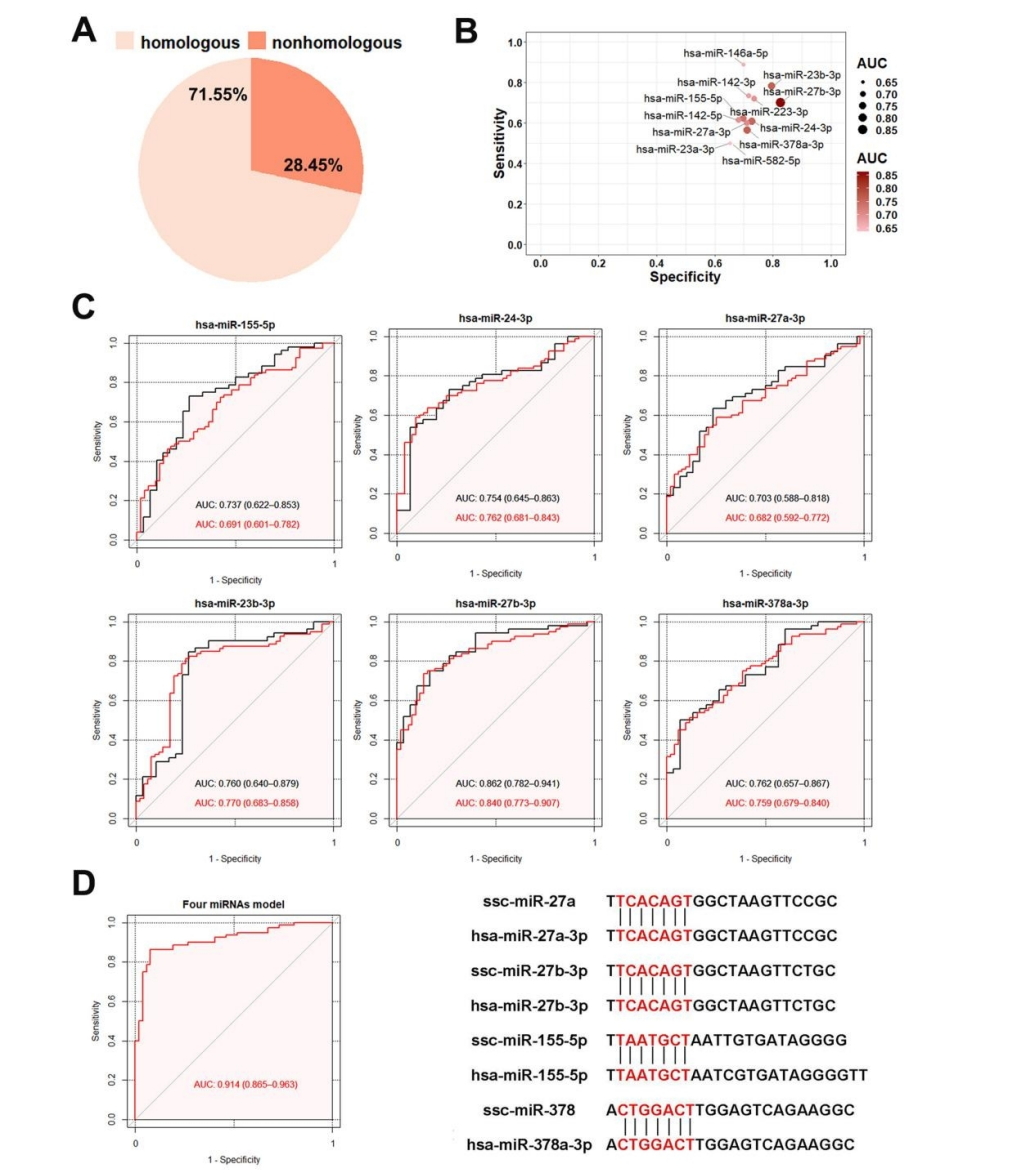
Comparison of miRNAs derived from SPEVs between pigs and humans. (A) Based on seed sequences of miRNAs, 71.55% were homologous between pigs and humans, and 28.45% did not show homology. (B) Twelve DEmis in the logistic module had a sensitivity of 0.5-0.9 and a specificity of 0.6-0.9. (C) ROC analysis of six DEmis in the test and validation sets (black is the test set, and red represents the validation set). (D) ROC analysis of the combination of four DEmis (hsa-miR-155-5p, hsa-miR-27a-3p, hsa-miR-27b-3p and hsa-378a-3p).

Based on seed sequences of miRNAs, most miRNAs (71.55%) were highly homologous between pigs and humans [Figure 8A]. We compared 27 DEmis detected in this study and found that 22 DEmis were highly conserved between pigs and humans. To avoid bias caused by some miRNAs with low expression levels, only 16 miRNAs (RPM > 1 in all samples) were included in the subsequent analysis. For the screening of potential biomarkers of PCa, we evaluated the specificity and sensitivity of each DEmi in the test set, which included 52 PCa cases and 30 controls. Our results suggest that most candidate DEmis displayed a sensitivity of 0.5-0.9 and a specificity of 0.6-0.9 [Figure 8B]. In addition, six miRNAs (hsa-miR-155-5p, hsa-miR-24-3p, hsa-miR-27a-3p, hsa-miR-23b-3p, hsa-miR-27b-3p, and hsa-miR-378a-3p) provided high AUC values (> 0.7) for discriminating between patients with PCa and controls. To validate the specificity of these six miRNAs in the test set, logistic regression and ROC analyses were performed using the validation set of 132 individuals, which included 80 PCa cases and 52 controls. hsa-miR-155-5p, hsa-miR-27a-3p, hsa-miR-27b-3p, hsa-miR-378a-3p, hsa-miR-24-3p, and hsa-miR-23b-3p exhibited AUCs of 0.691, 0.682, 0.840, 0.759, 0.762, and 0.770, respectively, which were close to the AUC values calculated from the test set [Figure 8C]. Additionally, four candidate miRNAs with high AUC values were combined using a logistic model, and better performance was obtained. hsa-miR-27a-3p, hsa-miR-27b-3p, hsa-miR-155-5p, and hsa-miR-378a-3p exhibited an AUC of 0.914 [Figure 8D], which was the highest AUC of all the combinations.

**Figure 9:**
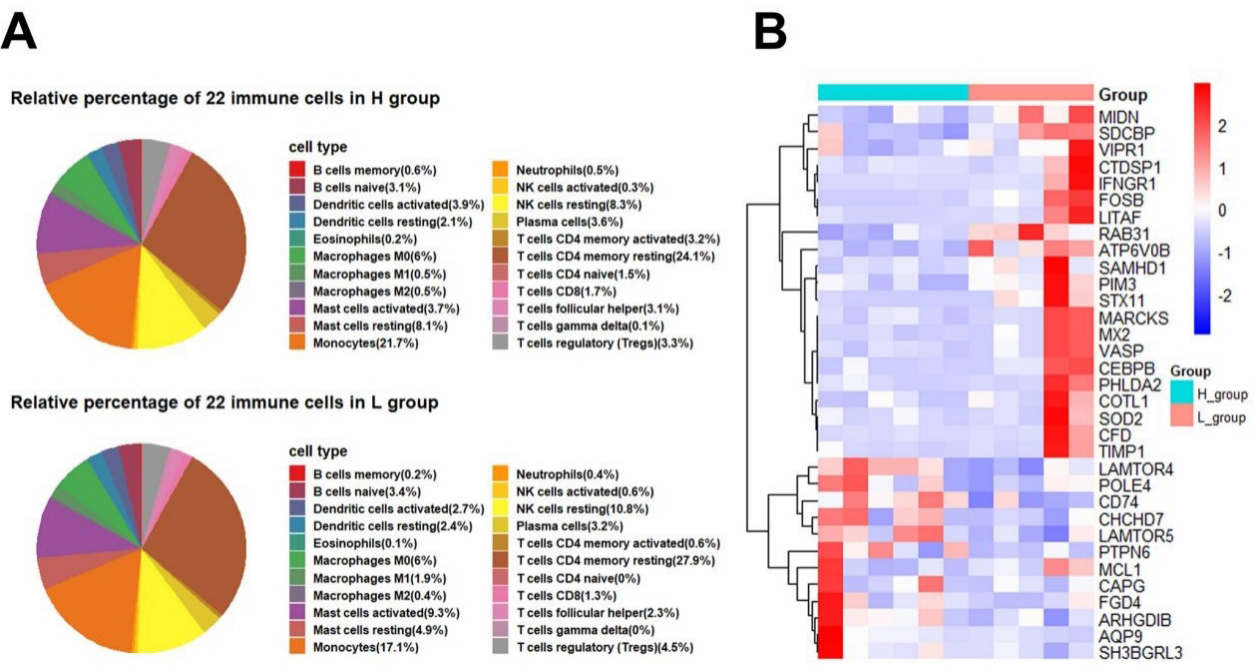
Proportion of different types of immune cells in SPEVs and expression of DEGs that overlap with immunocyte marker genes in the H and L groups. (A) Proportion of 22 immune cell populations in SPEVs of the H and L groups. (B) Heatmap of 30 DEGs that overlapped with monocytes marker genes.

Furthermore, we evaluated the mRNA composition of SPEVs using the CIBERSORTx website to identify the types and proportions of 22 immune cells based on cell markers. Interestingly, the proportions of different types of immune cells varied considerably, and we observed that resting memory CD4 T cells and monocytes were significantly enriched [Figure 9A]. Interestingly, we found that the percentage of resting memory CD4 T cells in the L group (27.9%) was slightly higher than that in the H group (24.1%), and the opposite results were found for monocytes, i.e., the percentage of monocytes in the L group (17.1%) was lower than that in the H group (21.7%). We also found that 33 DEGs detected in this study overlapped with the marker genes of monocytes, and most of these DEGs were upregulated in the L group [Figure 9B].

## DISCUSSION

It has been reported that SPEVs can combine sperm *in vitro* and affect sperm function, particularly sperm motility, in mammals^[55-57]^. Here, we isolated EVs from semen plasma of Yorkshire boars using an ultracentrifugation procedure and performed whole transcriptome sequencing of SPEVs from Yorkshire boars with high or low sperm motility. Some important miRNAs, genes and lncRNAs affecting sperm motility in SPEVs were identified in this study.

### Expression of miRNome in SPEVs of Yorkshire boars

A comparison of the small RNA libraries with different noncoding RNA databases revealed that most of the small RNAs in SPEVs were miRNAs. An analysis of the expression levels of these small RNAs showed that 83.71% of the miRNAs were expressed at low levels, and only 16 miRNAs were highly expressed in SPEVs. Among these miRNAs, five miRNAs (ssc-let-7c, ssc-let-7a, ssc-let-7f-5p, ssc-let-7e, and ssc-let-7i-5p) belonged to the let family. Similar results have been obtained in other studies. For example, let-7a, let-7c, let-7f, and let-7i were among the top 10 most abundant miRNAs detected in milk EVs of pigs^[58,59]^, cattle^[60]^, and humans^[59,61]^. let-7 is one of the earliest miRNAs discovered, and its family members are highly conserved in sequence and function. The let-7 family is reportedly related to human gamete differentiation and PCa^[62]^. let-7 plays an important role in mammals, such as participating in cell proliferation, differentiation, and apoptosis. Studies have shown that different members of the let-7 family are differentially expressed in the testis, sperm, and seminal plasma of patients with azoospermia, oligospermia, and asthenospermia^[25,63]^. In addition, previous studies have shown that miR-148a, miR-21-5p, and miR-125b are among the top 10 most abundant miRNAs in milk EVs and blood plasma EVs^[64,65]^. The high expression of these miRNAs in EVs from different tissues indicates that they might play an important role in the formation of EVs or some basic physiological function. However, we also found that some highly expressed miRNAs in SPEVs are different from those found in EVs derived from other tissues. For example, miR-10a, miR-10b, miR-125a, miR-16, and miR-26b-5p are only highly expressed in seminal plasma, which indicates that these miRNAs might play a particular role in the testis, epididymis, or sperm. It has been reported that miR-10b, miR-16, and miR-26b are related to PCa^[66-68]^. This finding also suggests the existence of differences in the types and contents of miRNAs in EVs, which might be related to the sorting of miRNAs during the formation of EVs. Many studies have found that some genes or proteins are related to the sorting of specific miRNAs into exosomes^[69]^. In this study, we also found the DEG *RAB31*, which was recently reported to control an ESCRT-independent exosome pathway in exosome biogenesis^[70]^. These results suggest that some DEGs in EVs might affect the sorting of miRNAs in EVs.

### Important DEmis, DEGs, and DELs related to sperm motility

In recent years, miRNAs have been recognized as key regulatory factors. It has been reported that miRNAs play an important role in sperm motility, azoospermia, and oligospermia. In the current study, a comparison of the H and L groups identified 27 DEmis, which included 18 important DEmis that were contained in the DEL-DEmi-DEG regulatory network. Interestingly, we observed two miRNA clusters, which included ssc-mir-23b, ssc-mir-24-2, and ssc-mir-27b and ssc-mir-23a, ssc-mir-24-1, and ssc-mir-27a, respectively. The positive correlation between the expression of the miRNAs in these two clusters was greater than 0.97 (*P *< 7.74 × 10^-7^ and* P *< 6.18 × 10^-7^, respectively). It has been reported that clustered miRNAs can coregulate and participate in many biological processes, such as metabolism, metabolic disorders, and cancer^[71,72]^. A previous study suggested that the expression of miR-23a, miR-23b, miR-27a, and miR-27b-3p in SPEVs of normal individuals was significantly different from that in SPEVs of individuals with oligozoospermia or asthenospermia^[24,73]^. Our results indicate that these miRNA clusters might play a synergistic role in regulating sperm motility.

Among the DEGs, ssc-miR-24-3p was predicted to be able to target and regulate 22 DEGs in the turquoise and blue modules, such as* MYC*, *MIDN*, *DDIT4*,* CTDSP1*,* ICAM1*, *CAPG*, and *LCN2*. ssc-miR-27b-3p can target *MKNK2* in the blue module and *PTPN6* in the red module. In addition, ssc-miR-23a and ssc-miR-23b simultaneously target *TMEM87A* in the brown module. Most of these genes were hub genes in the regulatory network and were found to be involved in HIF-1 signaling pathway, autophagy pathway in animals, MAPK signaling pathway, mTOR signaling pathway, Jak-STAT signaling pathway, TGF-beta signaling pathway, PI3K-Akt signaling pathway, Wnt signaling pathway, transcriptional misregulation in cancer, and microRNAs in cancer. It has been reported that *MYC* is highly expressed in diseased prostate tissues and is a very important gene associated with PCa^[74,75]^. Some studies have suggested that *MYC* cooperates with the dysregulation of the PI3K/AKT/mTOR pathway to promote PCa cell survival and promote oncogenic signaling in prostate cancer^[74]^. In addition, previous studies have shown that *DDIT4* is significantly downregulated in prostate cancer cells and that the induction of *DDIT4 *expression can regulate *MYC*, which is a downstream target of the mTOR signaling pathway^[76,77]^. Furthermore, *DDIT4* affects sperm motility through the autophagy pathway. Recent studies have shown that autophagy can degrade long-lived proteins and organelles and thus maintains the stability of spermatogenic cells, ensures sperm meiosis and spermatogenesis, and improves sperm motility^[78,79]^. However, high autophagy levels can also lead to the excessive consumption of protein and damage to organelles, which results in cell dysfunction and eventually leads to decreases in the number and motility of sperm^[80,81]^. The results of this study show that ssc-miR-1249, ssc-miR-1296-5p, and ssc-miR-24-3p act on mTOR, autophagy, and PI3K-Akt signaling pathways by targeting the *DDIT4* gene, respectively. Although these results remain to be confirmed, the findings suggest that miRNAs might target not only multiple components of a common pathway but also single components of different regulatory pathways.

lncRNAs can act as sponges of miRNAs to interact with miRNAs^[82]^. To explore the relationship between DELs and DEmis in SPEVs, we predicted the binding relationship between DELs and DEmis and calculated the correlation between the expression level of DELs and that of DEmis in the turquoise and blue modules. We found that 21 DELs and 5 DELs could target ssc-miR-24-3p in the turquoise and blue modules, respectively. Furthermore, our results show that the expression levels of five DELs (MSTRG.28673.8, MSTRG.39179.1, MSTRG.34347.1, MSTRG.52149.82, and MSTRG.37144.3) were significantly correlated with the expression level of ssc-miR-24-3p (correlation > 0.7, *P *< 0.05). Moreover, these DELs were closely related to *MYC*, *DDIT4*, *LCN2*, and *ICAM1 *in the turquoise and blue modules. Some studies have suggested that the RNA in sperm can be derived from SPEVs^[83]^. Therefore, we believe that these DEmis, DEGs, and DELs in SPEVs play an important role in sperm motility and function.

### Diagnosing human PCa by microRNAs derived from SPEVs

Fluid biopsy based on EVs is attracting increasing attention. Previous studies have shown that approximately 40% of semen is derived from prostatic tissue, and its contents are most likely to contain prostate disease-specific derived molecules, which can be potentially used as PCa biomarkers^[31]^. Due to the anatomical and physiological similarities between pigs and humans, pigs are increasingly regarded as an ideal model of human medicine. In this study, we found 16 DEmis that were highly conserved between pigs and humans by comparing the seed sequences of porcine and human miRNAs. Most of these are reportedly associated with PCa in humans. For example, miR-146a has been well studied in PCa, and several studies have shown that miR-146a inhibits the migration and invasion of PCa cells^[84]^. Several deregulated miRNAs in different liquid biopsies of PCa, such as mir-130a, mir-24, mir-223, and mir-155, have been reported^[31,66,68]^. Barcelo *et al*.^[31]^ (2018) found that hsa-miR-142-5p, hsa-miR-142-3p, hsa-miR-130a-3p, and hsa-miR-223-3p are highly expressed in seminal exosomes of patients with PCa.

Based on the human PCa data in the TCGA database, we constructed diagnostic models for the 16 DEmis and found that six DEmis (hsa-miR-155-5p, hsa-miR-24-3p, hsa-miR-27a-3p, hsa-miR-27b-3p, hsa-miR-23b-3p, and hsa-miR-378a-3p) have good predictive and diagnostic abilities (AUC > 0.7). Using a logistic model, four miRNAs (hsa-miR-27a-3p, hsa-miR-27b-3p, hsa-miR-155-5p, and hsa-miR-378a-3p) were combined, and the AUC value increased to 0.914. Our results suggest that pigs can be used as an ideal animal model for the study of human prostate diseases. Moreover, porcine SPEVs can be used as good research material to obtain valuable information of human male reproductive diseases.

### Close relationship between reproductive and immune characteristics

Recent studies have shown that the immune system is significantly related to a variety of reproductive traits^[85]^. In the present study, we found that some miRNAs in SPEVs are closely related to immunity in mammals. miRNAs are considered a critical regulatory molecule in the immune system^[86]^. It has been reported that miRNAs play an important role in the development, differentiation, and function of T cells and regulate general cellular biological processes in T cells, such as proliferation and apoptosis^[86,87]^. miR-155 is upregulated in B and T cells upon activation, and genetic gain- and loss-of-function studies have shown that miR-155 plays an important role in the control of germinal center reactions in vivo^[88-90]^. miR-146a exhibits a T cell subset-specific expression pattern and is involved in the processes underlying the regulation of specific T cell subsets^[91-93]^. The miR-17/92 cluster regulates T cell activation^[94,95]^. miR-17/92-deficient mice show increased pro-B cell apoptosis accompanied by a severe blockage of B cell development at the pro- to pre-B transition^[96]^. In contrast, the overexpression of miR-17/92 in mice results in the spontaneous activation and pronounced expansion of B and T lymphocytes^[97]^. In addition, the miRNA cluster consisting of miR-23a, miR-24, and miR-27a plays a critical role in the regulation of immune cell populations through the repression of B lymphopoiesis^[98]^.

To further identify specific types of immune cells associated with sperm, we explored the components of immune cells using the CIBERSORTx website based on the mRNA expression of SPEVs obtained in this study. We found that resting memory CD4 T cells and monocytes were mainly enriched. However, studies on the immune cell components of plasma EVs from normal subjects and patients with hepatocellular carcinoma have shown that neutrophils, M2 macrophages, and other natural killer cells are the most abundant in healthy individuals^[99]^. The types of immune cells in semen EVs are different from those in plasma, which indicates that the sources and functions of immune cells in different types of EVs might be different. Additionally, we found that 33 DEGs were also marker genes of monocytes, and 30 of these genes, such as *RAB31*, *ATP6V0B*, *CHCHD7*, *LAMTOR5*, and *PTPN6*, were also hub genes in the DEL-DEmi-DEG regulatory network and can be considered important candidate genes for further verification.

In conclusion, the present study provided a comprehensive analysis of the whole transcriptome of Yorkshire boar SPEVs and revealed several important miRNAs, genes, and lncRNAs in SPEVs associated with sperm motility. hsa-miR-155-5p, hsa-miR-23b-3p, hsa-miR-27a-3p, hsa-miR-27b-3p, hsa-miR-24-3p, and hsa-miR-378a-3p might be used as promising biomarkers for the diagnosis of PCa. In addition, we found that reproductive traits exhibit a close relationship with immune traits and that resting memory CD4 T cells and monocytes are enriched in SPEVs. The results obtained in this study provide a new perspective and better understanding for further study of sperm motility in male mammals.

## DECLARATIONS

### Copyright

© The Author(s) 2021.
