# Erratum: Identification of important extracellular vesicle RNA molecules related to sperm motility and prostate cancer

**DOI:** 10.20517/evcna.2023.35

**Published:** 2023-07-11

**Authors:** EVCNA Editorial Office

**Affiliations:** Xi'an Office, Suite 1504, Tower A, Xi'an National Digital Publishing Base, No. 996 Tiangu 7th Road, Gaoxin District, Xi'an 710077, Shaanxi, China.



The Editorial Office wants to make the following corrections to this paper^[1]^.

During the production stage, the Ethical approval and consent to participation were wrongly stated in the DECLARATION section, while the authors described the Ethics statements in the METHODS section appropriately. The Ethical approval and consent to participate should be “All protocols for the collection of semen samples of all animals were approved by the Institutional Animal Care and Use Committee at China Agricultural University (Permit number: DK996). The experiments in this study were conducted according to regulations and guidelines established by this committee.”

We apologize for any inconvenience caused and state that the scientific conclusions are unaffected. The original article has been updated.

## References

[1] Zhang Y, Ding N, Xie S (2021). Identification of important extracellular vesicle RNA molecules related to sperm motility and prostate cancer. Extracell Vesicles Circ Nucleic Acids.

